# Laceration of the Cervix Uteri

**Published:** 1856-10

**Authors:** J. R. Freese

**Affiliations:** Bloomington, Ill., Professor elect of Surgery, in the Western Law and Medical College


*Laceration of the Cervix Uteri. From Preternatural Rigidity.* By J. R. Freese, M. D., of Bloomington, Ill., Professor elect of Surgery, in the Western Law and Medical College.

A case of this character fell under my care a few days ago; and, as it is a *rare* condition of things, and is liable to give a good deal of *anxiety* to the uninitiated, I have concluded to report it for the benefit of my younger medical brethren.

On the 22d of May last, I was called to attend Mrs. C. who was then in labor with her second child—the first having been born about two years previous. Upon examination, per vaginam, I found *two* distinct openings into the womb—one anterior to the other. I passed my finger in at the one, and out at the other; hooking the lacerated part over the first phalange of the index finger. The septum, or lacerated part, seemed to be nearly a half inch broad, by an eighth in thickness. Upon a still closer examination, I found the posterior opening to be the natural *os tincæ*, while the anterior was the lacerated opening. The presentation was natural; and, as the labor progressed, I found the anterior opening more dilated than the posterior, and supposed that the fœtus would emerge through that opening. I hesitated for a few moments, whether to draw the septum forward, and thus try to have the child pass through the natural os, lest the other one might be still further lacerated, or let it take its own course, and finally decided upon the latter. If, thought I, the rigidity of the os was too great in the first instance, it is probably too great still; besides, the labor would be greatly retarded, as the natural os was so far posterior that the vertex would press unduly against the sacrum, and thus the fœtus would be thrown out of Carus' curve, the line of which seemed to correspond with the lacerated opening.

The labor continued about six hours, when a "man-child" was born into the world—which unfortunately, however, proved to be "before its time," probably about a seven or eight month's child. It was with some difficulty that I established respiration—every care was taken, but the child lived only about twelve hours.

The expulsion of the placenta was arrested by the septum, and I had to pass my hand into the vagina to release it, before it could be brought away.

The woman had a strong disposition to peritoneal inflammation; but, by timely assistance, this was prevented, and she was "up and about" in nine days.

I learned from her that, in her first confinement, she had very strong and long continued pains, but that "*suddenly*," to use her own expression, the child was born. Her medical attendant gave her no reason for it, and the probabilities are that he mistrusted not the undue rigidity, and knew nothing of the rupture in the cervix uteri. Her convalescense was tedious, and she still suffered more or less from weakness, pains in the back, an uneasy sensation about the womb, etc.

The *rationale* of the case I presume to be, that, previous to her first gestation she had *anteversion* of the womb, together with *extreme rigidity* of the os uteri, both of which conditions continued to a certain extent, up to the time of her confinement. Then, the mouth of the womb, resting against the sacrum, together with this extreme rigidity, prevented the child from being born *per viæ naturales*, and hence the rupture, *anterior* to the mouth, through which the child was "suddenly" born. This rupture finally healed at its edges, leaving, however, a *double* opening into the womb—through the false one of which the last child was born, and all subsequent ones will be, if she continues in the "good old way."

This was not a case of *occlusion* of the os uteri, as I could readily pass my finger in or out of the natural os. Neither do I think that it depended on *obliquity* alone, as Velpeau, Baudelocque, Desormeaux, North and others think that many cases of ruptured womb do; but it was a consequence, in my opinion, of the two abnormal conditions combined, viz: obliquity and rigidity.

If her medical attendant, in her first confinement, had known the exact condition of parts, perhaps he might have prevented the rupture, by elevating and supporting the fundus of the womb—drawing forward the mouth, administering nauseating dose of tartarized antimony with opium, bleeding, *ad diliqueum animi*, if necessary, or, even incising, with a probe pointed bistary, one side of the cervix uteri; but, as he knew it not, and did nothing, Nature took the case in her own hands. made and an incision in the "natural way," and left two openings instead of one, into the great *matrix materni*—that wonderful "fountain of life!"

June 18th, 1856.

